# Introduction of novel intravascular ultrasound preceding with angled guiding catheter (I-PAD) technique to treat chronic total occlusions in peripheral artery disease

**DOI:** 10.1186/s42155-024-00469-z

**Published:** 2024-07-11

**Authors:** Mitsuo Sobajima, Teruhiko Imamura, Yohei Ueno, Hiroshi Onoda, Ryuichi Ushijima, Hiroshi Ueno, Koichiro Kinugawa

**Affiliations:** The Second Department of Internal Medicine, Graduate School of Medicine, University of Toyama2630, Sugitani, Toyama, Japan

**Keywords:** Novel technique, Intravascular ultrasound, Angled guiding catheter, Endovascular treatment, Peripheral artery disease, Chronic total occlusion

## Abstract

**Background:**

The optimal endovascular treatment (EVT) for chronic total occlusion (CTO) lesions in patients with peripheral artery disease (PAD) has remained unestablished. We encountered a patient with PAD in whom CTO was successfully treated using a novel technique that involved intravascular ultrasound (IVUS) and angled guiding catheter: IVUS Preceding with Angled guiDing catheter (I-PAD) technique.

**Case presentation:**

A 74-year-old male presented with intermittent claudication attributed to CTO of the right external iliac artery. EVT was performed via the right common femoral artery. We retrogradely advanced the I-PAD system (i.e. partially extending the IVUS transducer portion from the tip of the angled guiding catheter) in the CTO lesion under the real-time guidance of IVUS imaging. We successfully traversed the CTO lesion without the use of a guidewire in approximately three minutes. The procedure concluded successfully without any procedure-related complications, following optimal stenting.

**Conclusions:**

The I-PAD might be an effective technique to accurately, quickly, and safely pass through CTO lesions.

**Supplementary Information:**

The online version contains supplementary material available at 10.1186/s42155-024-00469-z.

## Background

The optimal endovascular therapeutic strategy for chronic total occlusion (CTO) lesions in patients with peripheral artery disease (PAD) has not yet been established, although several procedural techniques have been employed by each operator [1–5]. Here, we propose a novel technique for passing CTO lesions by combining an intravascular ultrasound (IVUS) and angled guiding catheter without a guidewire named “IVUS Preceding with Angled guiDing catheter (I-PAD) technique”.

## Case presentation

**Fig. 1:**
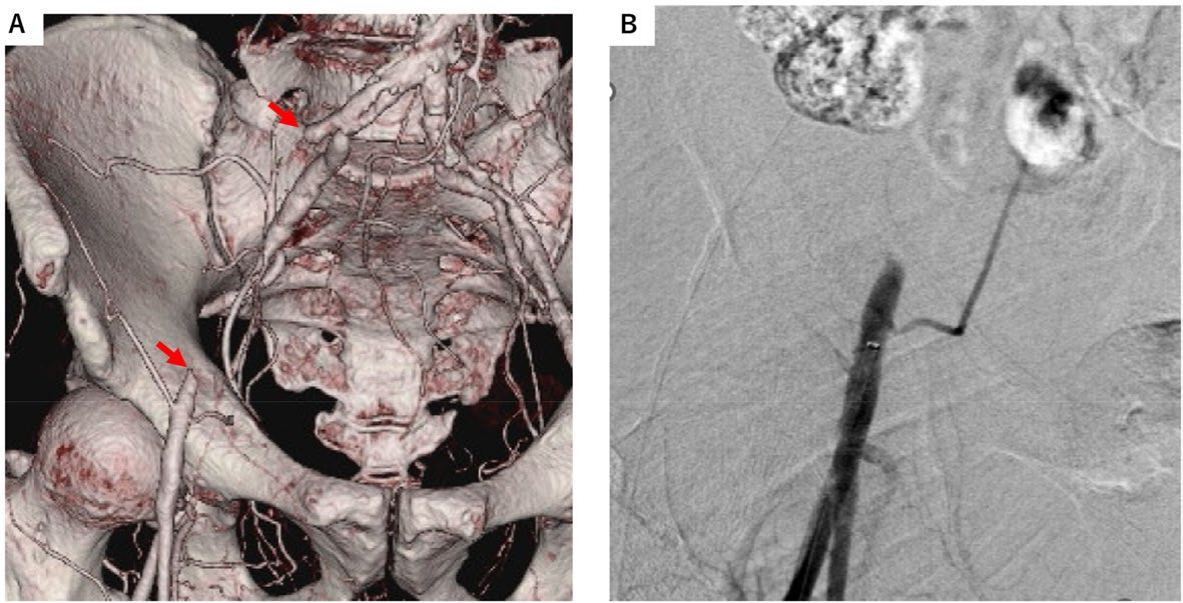
The findings of CTO lesion by enhanced 3-dimensional computed tomography (**A**) and initial angiography (**B**)

A 74-year-old male presented with intermittent claudication (Rutheford category III) in his right lower limb. Contrast-enhanced computed tomography displayed CTO in his right external iliac artery (Fig. 1A). We scheduled endovascular treatment (EVT) for his CTO.

**Fig. 2:**
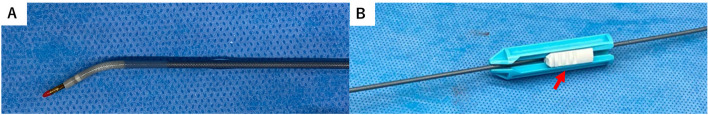
I-PAD system; Partially extending the IVUS transducer portion from the angled guiding catheter tip (**A**) fixing IVUS and angled guiding catheter with the torque device (**B**). Aligning the direction of the angled guiding catheter's tip with the position of the torque device’s stopper (red arrow) makes it easier to determine the orientation of the catheter tip

We retrogradely accessed the 6-Fr guiding sheath via his right common femoral artery and performed initial angiography (Fig. 1B). The I-PAD system was established by partially extending the IVUS (Eagle Eye Platinum short-tip, Philips Volcano, CA, USA) transducer portion from the tip of the angled guiding catheter (GoGo catheter D-shape, Medikit, Tokyo, Japan) and fixing them together with a torque device (Namic The Grip, Medline Industries, IL, USA) (Fig. 2A, B).

**Fig. 3:**
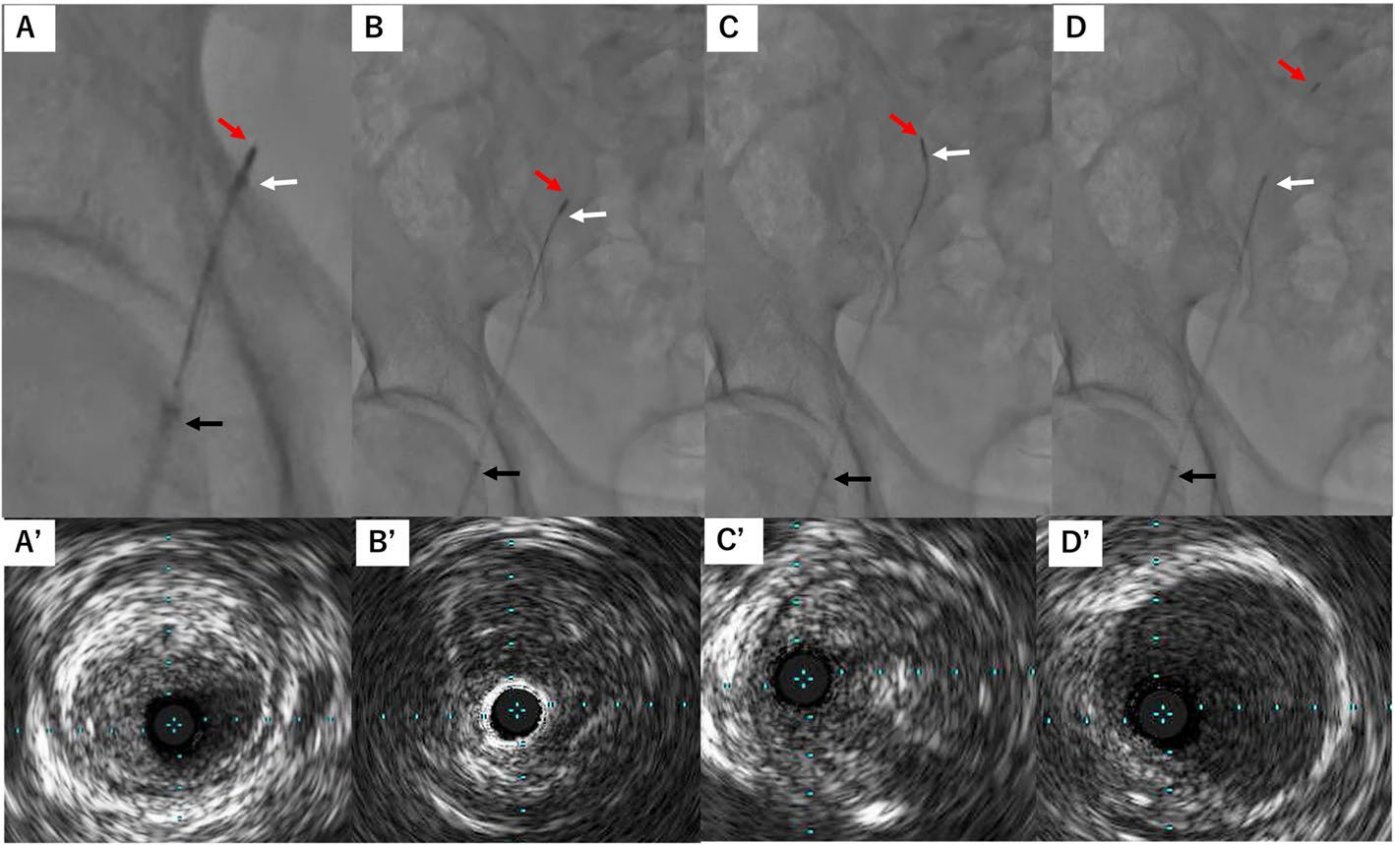
The tip of the guiding sheath (black arrow), the tip of the angled guiding catheter (white arrow), and the tip of the IVUS (red arrow). Fluoroscopic image (**A**) and IVUS image (A’) at the point where the I-PAD system has retrogradely advanced to the central of the occluded external iliac artery. Fluoroscopic image (**B**) and IVUS image (B’) at the point where the system is further advanced in the external iliac artery from position A. Fluoroscopic image (**C**) and IVUS image (C’) at the point where the system advanced deviated toward the vessel wall. Fluoroscopic image (**D**) and IVUS image (D’) at the point where the system was slightly retracted and directed toward the center of the vessel and only IVUS passed to the common iliac artery

Under the real-time guidance of IVUS imaging, the tip of the system was advanced in the CTO lesion of the external iliac artery without a guidewire (Fig. 3A). We carefully advanced the system within the CTO lesion, directing the tip toward the center of the vessel and avoiding proximity to the vessel wall (Fig. 3B).

**Fig. 4:**
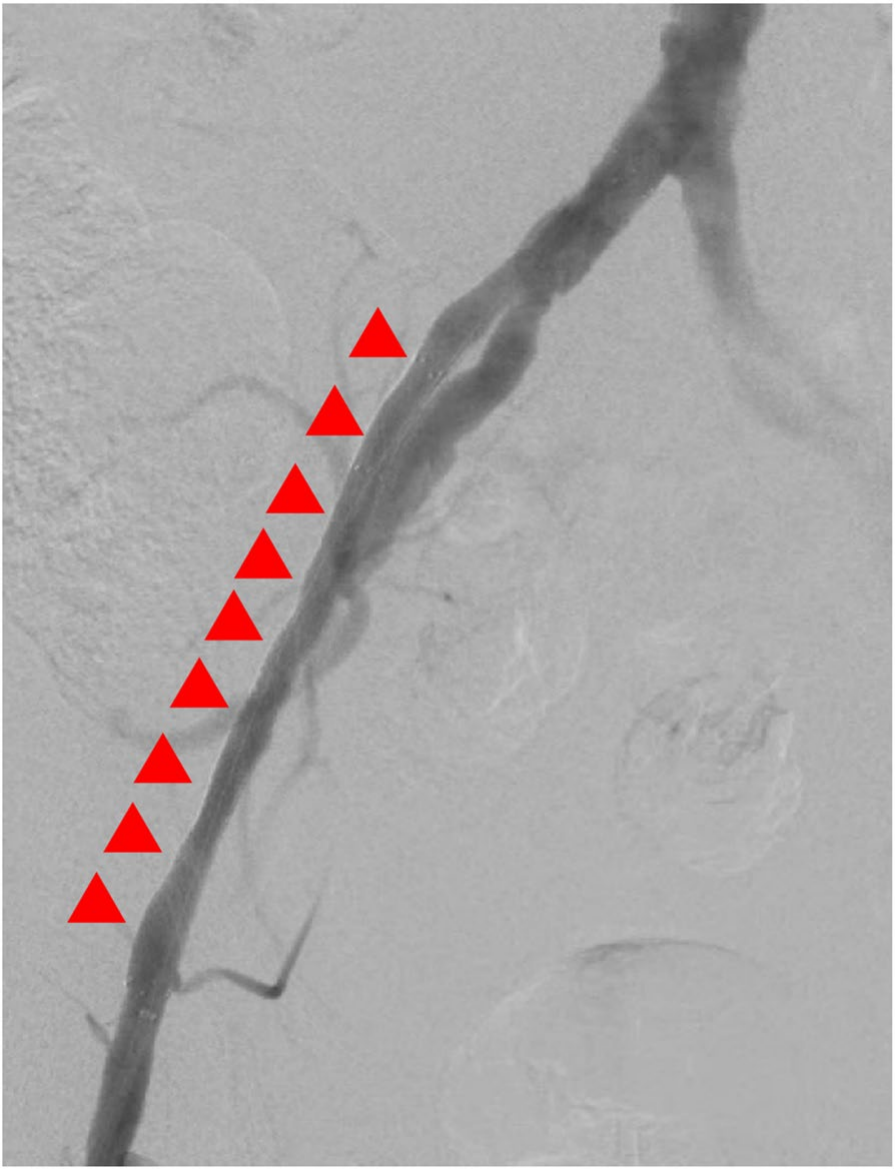
The findings of final angiography after stenting. The red triangles indicate the stent

When the tip deviated toward the vessel wall (Fig. 3C), we retracted the system slightly and reoriented the tip towards the vessel center, successfully passing the CTO lesion preceding IVUS alone in approximately three minutes. (Fig. 3D, Supplementary Video). After passing through the CTO lesion, we advanced a guidewire and removed the angled guiding catheter, followed by the appropriately-sized balloon dilatation based on the IVUS findings. The procedure concluded successfully without any procedure-related complications through optimal stenting (Fig. 4).

## Discussions

The Eagle Eye Platinum short-tip IVUS features a relatively stiff 0.019-inch tip with a nose cone design. Its maximum outer diameter is 0.046 inches, fitting snugly into the 0.062-inch inner diameter of the GoGo catheter D-shape. This configuration offers robust backup support for advancing the system in the appropriate direction within CTO lesions. The IVUS transducer is located only 2.5 mm proximal from the tip, facilitating safe real-time monitoring of the tip of the system during the procedure.

In detail, we use two types of technique during the procedure: (1) a direct advancement with an angled guiding catheter together with IVUS (Suppl. A–D) and (2) an indirect advancement using the angled guiding catheter for directional control while advancing IVUS alone (Suppl. E–G).

(1) The former technique provides strong backup force and controllability, making it effective for advancing through large-size tortuous vessels like the iliac artery. (2) The latter technique has a reduced backup force compared to the former one, whereas it may be useful for advancing through middle-size straight vessels like the superficial femoral artery. The choice between these techniques depends on the vessel's features, including the vessel’s diameter, straightness, and plaque hardness. If both techniques fail to advance the IVUS, we can insert a 0.014-inch wire into the GoGo catheter, enabling us to switch directly to the conventional IVUS guide wiring technique [4].

Another group also reported a relatively similar technique using IVUS and the GoGo catheter to treat CTO [5]. However, there are several obvious differences between the two methods in how IVUS is advanced and the type of GoGo catheter. The previous report advanced IVUS independently from the “straight-type” GoGo catheter. Once IVUS went into a false lumen, they needed to switch to wire manipulation because the direction of IVUS could not be controlled. Conversely, our approach primarily involves advancing IVUS together with an angle-type GoGo catheter “fixed” by a torque device. This allows us to control the direction of IVUS advancement and redirect towards the central vessel axis if necessary. Thus, the I-PAD technique should enable real-time visualization of the system’s tip, reduce the risk of vessel perforation, construct consistent intraplaque passage for optimal balloon dilatation, decrease procedural time and radiation exposure, minimize contrast agent use, and save unnecessary device usage leading to cost savings. Given our accumulating experiences including the present case, if we confirm non-calcified CTO lesions in the supra popliteal artery by contrast-enhanced CT preoperatively, we highly recommend this technique. Conversely, this technique is not recommended in cases of calcified lesions or small vessels less than 5 mm in diameter, where manipulation with the I-PAD system is challenging.

## Conclusion

The I-PAD technique offers a promising approach for treating CTO in PAD patients. Further studies are warranted to validate its efficacy and feasibility.

### Supplementary Information

Supplementary Material 1: Supplementary figure. Schema of technique. To advance the I-PAD system securely within occluded vessels (A), if deviation towards the vessel wall occurs (B), the system is gently retracted (C) and redirected towards the center of the vessel (D). In addition to repeating steps A to D, there is an alternative step like E to G. IVUS can be advanced alone apart from the angled guiding catheter (E). If the IVUS deviates towards the vessel wall (E), an angled guiding catheter is brought closer to the IVUS (F) and redirected toward the center of the vessel (G).  In instances like C and F, where the IVUS deviates towards the vessel wall, fluoroscopy is rotated to a view where the shape of the angled guiding catheter is discernible, enabling adjustment of the guiding catheter tip opposite to the deviated IVUS direction, thereby redirecting towards the center of the vessel.

Supplementary Material 2: Supplementary video. This video synchronizes IVUS and fluoroscopic images during the IPAD technique shown in Fig. 3.

## Data Availability

The data and material on the case report are available from the corresponding author, MS, upon reasonable request.

## Abbreviations

I-PAD: Intravascular ultrasound preceding with angled guiding catheter
EVT: Endovascular treatment
CTO: Chronic total occlusion
PAD: Peripheral artery disease
IVUS: Intravascular ultrasound
