# Supplementary figures and images for: Introduction of novel intravascular ultrasound preceding with angled guiding catheter (I-PAD) technique to treat chronic total occlusions in peripheral artery disease
Source: CVIR Endovasc. 2024 Jul 11;7:54. doi: 10.1186/s42155-024-00469-z (PMC11239645; doi:10.1186/s42155-024-00469-z)

## Slide 1
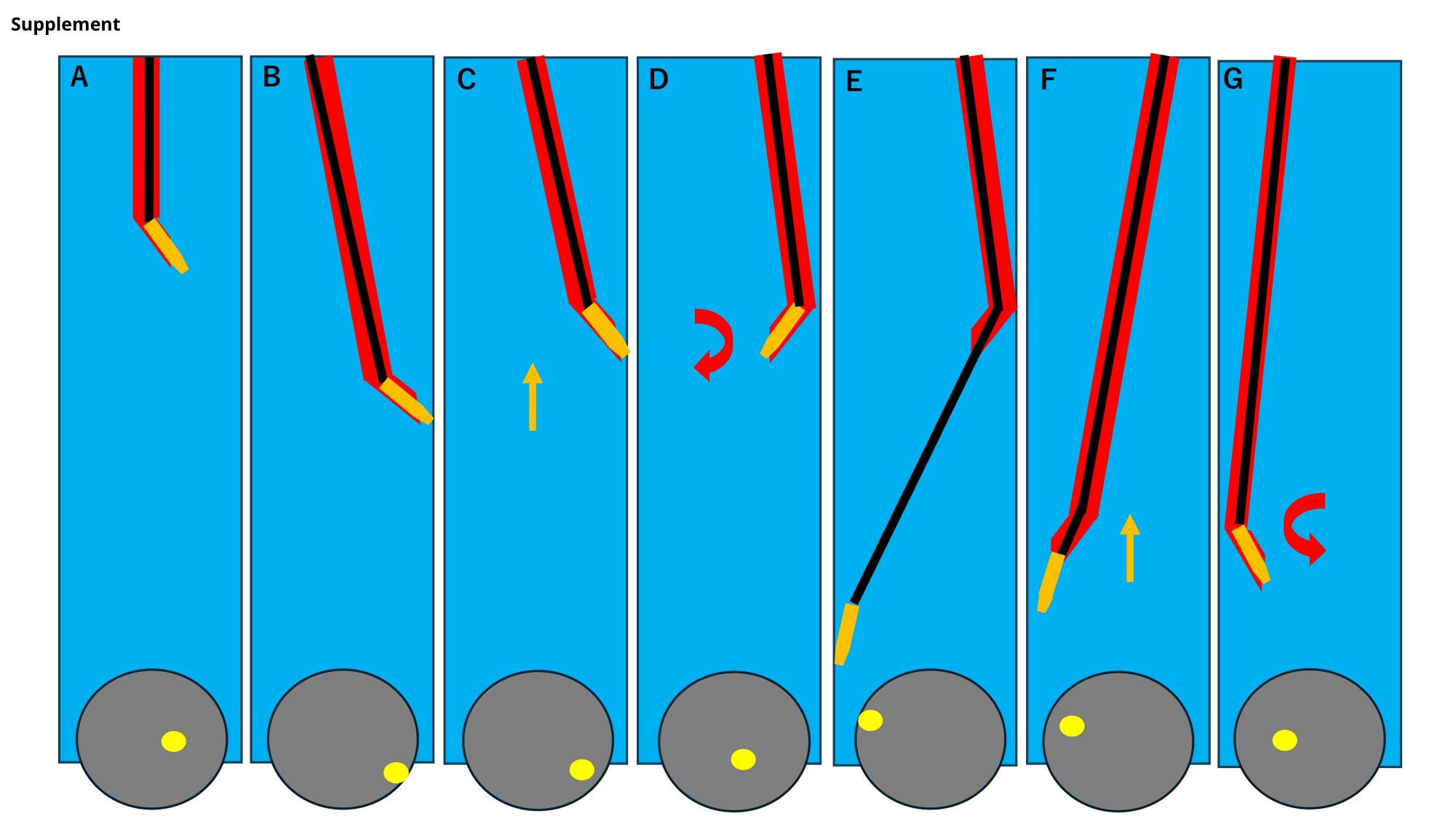

Supplement

Supplement: Supplementary file 1 — Supplementary Material 1: Supplementary figure. Schema of technique. To advance the I-PAD system securely within occluded vessels (A), if deviation towards the vessel wall occurs (B), the system is gently retracted (C) and redirected towards the center of the vessel (D). In addition to repeating steps A to D, there is an alternative step like E to G. IVUS can be advanced alone apart from the angled guiding catheter (E). If the IVUS deviates towards the vessel wall (E), an angled guiding catheter is brought closer to the IVUS (F) and redirected toward the center of the vessel (G). In instances like C and F, where the IVUS deviates towards the vessel wall, fluoroscopy is rotated to a view where the shape of the angled guiding catheter is discernible, enabling adjustment of the guiding catheter tip opposite to the deviated IVUS direction, thereby redirecting towards the center of the vessel. [file 42155_2024_469_MOESM1_ESM.pptx]
